# Self-reported Body Changes and Associated Factors in Persons Living with HIV

**DOI:** 10.3329/jhpn.v28i6.6604

**Published:** 2010-12

**Authors:** Kelly Virecoulon Giudici, Ana Clara F.L. Duran, Patricia Constante Jaime

**Affiliations:** Department of Nutrition, School of Public Health, University of São Paulo, São Paulo, Brazil

**Keywords:** Body-weight, Cross-sectional studies, Diet, HAART, HIV, Perceptions, Brazil

## Abstract

The study aimed at verifying the associated factors of self-perceived body changes in adults living with HIV in highly-active antiretroviral therapy (HAART) in the city of São Paulo, Brazil. This cross-sectional study was conducted among people living with HIV on HAART for at least three months. A standardized questionnaire was used for assessing self-perceived body changes. Associated factors relating to self-reported body changes in people living with HIV (PLHIV) were assessed with Student's *t*-test and chi-square test. In total, 507 patients were evaluated. The mean time since diagnosis was 6.6 years [standard deviation (SD)±4.1], and the mean duration of HAART was 5.1 years (SD±3.3). Self-perceived body changes were reported by 79.5% of the participants and were associated with viral load and duration of HAART. Fibre intake was lower among males who gained in abdominal fat (p=0.035). HAART-related body changes were reported by the large majority of the population and were associated with demographic and clinical variables.

## INTRODUCTION

Brazil, being the most populated country in Latin America, also holds one-third of all AIDS cases in the region. During 1980–June 2009, 544,846 cases of AIDS were noticed, corresponding to an incidence rate of 18.2 per 100,000 inhabitants (1). Of the noticed cases, 65.4% were men, and 34.6% were women. According to the United Nations Joint Programme on HIV/AIDS (UNAIDS), the number of infected people in the country varies from 600,000 to 890,000 (2). São Paulo—the biggest city of Brazil—has the largest number of people living with HIV (PLHIV) in the country.

In developed countries, the tendency of reduction in mortality due to AIDS had been observed even before the appearance of the highly-active antiretroviral therapy (HAART), and this has been attributed to the prophylaxis and better clinical treatment of opportunistic infections. However, with the use of protease inhibitors, this phenomenon was accentuated (3).

Ever since universal access to HAART through the public-health system in Brazil (in 1996), the country has become an example for the treatment of those living with HIV. The public-healthcare programme for PLHIV also includes other initiatives aimed at reducing hospital admissions, such as specialized outpatient care, day hospitals, and home-based care (3). Consequently, the rate of HIV infection-related mortality has declined from 9.6 per 100,000 inhabitants in 1996 to 6.1 per 100,000 inhabitants in 2008, resulting in increases in survival (1).

With great progress in the clinical course, prognostic and survival of patients, HIV infection starts to be seen like a disease of chronic evolution and potentially controllable (4). The prolonged use of HAART has led to side-effects which have brought new demands to health services specialized in the care of PLHIV (5).

Lipodystrophy syndrome, defined as a set of changes that includes loss of fat in peripheral areas, such as face, buttocks, arms, and legs, and gain in fat in central portions of the body, such as abdomen, neck, and chest and in the arms, is currently considered one of the most important side-effects of the use of HAART that may lead to other problems relating to stigma towards PLHIV (6). In a study among HAART users, perceived body changes were significantly associated with the duration of the use of protease inhibitors and nucleoside reverse transcriptase inhibitors (6).

The fact that not all patients on HAART develop fat redistribution, dyslipidaemia, or impaired glucose homeostasis supports the hypothesis that the susceptibility to morphologic and metabolic changes varies by therapy, environment, and genetics (7). In a study on self-reported body changes, Santos *et al*. found a prevalence of 49.2% of self-perception of gain in abdominal fat in PLHIV in Brazil (6). Overweight and obesity are turning into a major public-health concern in the country, reaching 45% of Brazilian adults in 2003 (8). The rates of obesity among men and women increased by 92% and 63% between 1975 and 1989 respectively, and between 1989 and 2003, a further rise was observed—10% among women and 26% among men, even higher among lower-income groups (9).

The role of an adequate intake of fibres in the prevention of diseases is already well-known (10). Benefits, among others, included reduction of lipid concentrations in blood, better tolerance to glucose, reduction of hyperinsulinaemia, control of body-weight, better gastrointestinal function, and reduction in the risk of cardiovascular diseases. Therefore, this study aimed at verifying the associated factors relating to self-reported body changes in PLHIV receiving HAART in the city of São Paulo, Brazil.

## MATERIALS AND METHODS

### Sample

Data from participants of this cross-sectional study were collected in nine of 15 STD/AIDS Specialized Health Community Centres of the City Council STD/AIDS Programme. These health community centres were located at four different geographic regions of the city of São Paulo, and there were no differences in the socioeconomic characteristics when the included health centres were compared with those excluded due to infrastructural problems.

Adults, aged 20–59 years, living with HIV, who had been on HAART for at least three months, were invited to join the study. Pregnant women and those who had undergone aesthetic surgery in the last six months (except for facial fill surgery) were excluded. Participants were chosen using a consecutive cumulative approach during November 2007–June 2008.

### Collection of data

A standardized questionnaire was used for collecting demographic data on age and schooling. Clinical data, which included CD4 cell count (cells/mm^3^) analyzed by flow cytometry (FACS Calibur, BD) and viral load (copies/mL) measured by branched DNA assay (Versant 440, Siemens), were obtained from the medical records of the participants, with a maximum time interval of six months before and after the interview day. Viral load was analyzed as a dichotomous variable with a considered threshold of 50 copies/mL. Information on the duration of known HIV infection and of HAART-use was also obtained from the medical records of the participants.

Body mass index (BMI) was used for characterizing the nutritional status of the study population, and it was estimated from corrected measures of height and weight. The methodology for the study has been validated and published elsewhere (11).

Of 540 persons interviewed, 32 were excluded because they were considered important outliers (below percentile 3 and above percentile 97 for the total energy intake). One participant was excluded from the sample because he did not answer all questions regarding self-perception of body changes. Data on 507 individuals were analyzed. The sample-size for the analyses of CD4 cell count and viral load was 501 and 473 respectively due to missing values.

Detailed description of types of foods and amount consumed the day before the interview was obtained using a rigorous standardization and was included individually in the nutritional software NutWin (DIS-EPM, São Paulo, Brazil). Standardizations were based on Brazilian guides (12, 13).

Dietary variables studied were: energy, carbohydrate, fibre, protein, total fat, and saturated fat. Fibre intake was corrected by the total energy intake using the residual method (14).

Reported self-perception of body changes was defined based on the answer provided to the question, addressing the self-perception of an increase or reduction of whatever severity in at least one specific body part, including face, buttock, arms, legs, neck, chest, and abdomen.

The sample was divided into two groups: those who reported any gain in body fat or loss of fat or lean mass, and those who did not report any body change. Frequency of self-reported body changes was described by the percent distribution of each of the cited groups of distinct patterns of change stratified by gender. Such groups were compared by demographic factors (schooling and age) and clinical factors (TCD4+ cells, viral load, time since diagnosis of HIV, and duration of HAART).

BMI and food intake of the study population (energy, macronutrients, saturated fat, dietary fibre, and density of fibre intake—dietary fibre (g)/1,000 kcal) were compared according to the presence of any perceived body change; any perceived gain in body fat; and any perceived fat or lean mass loss.

After interviews, all the participants received brief nutritional counselling for promoting healthful eating, physical activity, and prevention of biochemical and morphological changes, in agreement with the recommendations of the Dietary Guidelines for the Brazilian Population (15) and Nutritional Recommendations to Individuals Living with HIV/AIDS (16). They were also referred to dietitians of the STD/AIDS Specialized Health Community Centres, if necessary.

### Analysis of data

Student's *t*-test was used for continuous variables, and categorical variables were analyzed by Pearson's chi-square test. 95% confidence interval (CI) was carried out to estimate the significant prevalence of self-reported body changes. Associations between the mean dietary fibre intake and the self-reported body changes were assessed with the Student's *t*-test. Statistical analyses were performed using the SPSS software (version 13.0) (SPSS Inc., Chicago, IL, USA).

### Ethics

Patients who agreed to participate were admitted after they signed an informed consent form and were fully informed about the objectives and procedures of the research. The Ethics Committees of both School of Public Health of the University of São Paulo and Health Secretariat of the municipality of São Paulo approved the study. Anonymity of subjects and confidentiality of information were ensured at all times.

## RESULTS

In total, 507 patients were evaluated, and their mean age was 41.7 years [standard deviation (SD)±7.8], of which 57.8% were male. The mean time since diagnosis was 6.6 years (SD±4.1), and the mean duration of antiretroviral treatment was 5.1 years (SD±3.3) (Table 1).

**Table 1.** Characteristics of the study sample. São Paulo, Brazil, 2008

Characteristics	Total		Male		Female	
	No.	%†	No.	%†	No.	%†
Age (years)‡						
20–29	33	6.5	18	6.1	15	7.0
30–39	182	35.9	96	32.8	86	40.2
40–49	210	41.4	124	42.3	86	40.2
>50	82	16.2	55	18.8	27	12.6
Schooling (years)‡						
<8	274	54.2	148	50.7	126	58.9*
8–11	173	34.2	99	33.9	74	34.6
>11	59	11.7	45	15.4	14	6.5
TCD4+ cells (cells/mm^3^)‡						
<200	86	17.2	49	16.8	37	17.6
200–499	222	44.3	138	47.4	84	40.0
≥500	193	38.5	104	35.7	89	42.4
Viral load‡						
Undetectable	137	29.0	76	26.9	61	32.1
Detectable	336	71.0	207	73.1	129	67.9
BMI‡						
<18.5	26	5.4	7	2.5	19	9.8*
18.5–24.99	288	60.3	178	62.5	110	57.0
25–29.99	130	27.2	87	30.5	43	22.3
≥30	34	7.1	13	4.5	21	10.9
Time since HIV diagnosis (years)¶, $	6.61	4.08	6.35	4.14	6.97	3.97
Duration of HAART (years)¶, $	5.11	3.30	5.10	3.27	5.12	3.35

*p<0.05;

†Except where indicated otherwise;

‡Pearson's chi-square test;

¶Student's t-test;

$Mean (standard deviation);

BMI=Body mass index;

HAART=Highly-active antiretroviral therapy

Of the participants, 54.2% had eight or less years of schooling, and women had lower schooling rates than men (p=0.007). Of the men (n=293), 35.0% were considered overweight (BMI ≥25) while 33.2% of the women (n=214) had a BMI of ≥25. More women than men were considered underweight (p<0.001).

Of the study subjects, 403 (79.5%) reported self-perception of body changes after the initiation of HAART. Loss of body fat or lean mass was reported by 54.6% [confidence interval (CI) 95% 50.3–59.0] of the patients, and 59.2% (CI 95% 54.9–63.5) reported gain in body fat. The self-perceived prevalence of body changes was higher among women (83.6%, CI 95% 78.6–88.6) compared to men (76.5%, CI 95% 71.6–81.3) (p=0.048). Gain in abdominal fat was perceived by 53.5% of the participants.

Those who reported any body change had been receiving HAART for a longer period than those who did not (p=0,035) (Table 2). Of the participants who reported gain in fat or loss of fat or lean mass in at least one body part, 31.6% presented undetectable viral load while the same was seen in 18.7% of those who did not perceive it (p=0.013).

**Table 2.** Clinical and demographic characteristics of adults living with HIV according to self-perception of HAART-related body changes, São Paulo, Brazil, 2008

Characteristics	Any body change (gain in fat or loss in fat or lean mass)			
	Yes		No	
	No.	%†	No.	%†
TCD4+ cells (cells/mm^3^)‡				
<200	68	17.1	18	17.3
200–499	180	45.4	42	40.4
≥500	149	37.5	44	42.3
Viral load (copies/mL)‡				
Undetectable	119	31.6	18	18.7*
Detectable	258	68.4	78	81.3
Time (years) since diagnosis¶, $	6.68	4.04	6.33	4.23
Duration (years) of HAART¶, $	5.27	3.39	4.47	2.85*
Age (years)¶, $	41.90	7.77	41.02	8.07
Schooling (years)¶, $	8.27	3.65	8.19	4.05

*p<0.05;

†Except where indicated otherwise;

‡Pearson's chi-square test;

¶Student's *t*-test;

$Mean (standard deviation);

HAART=Highly-active antiretroviral therapy

The mean BMI was higher among persons who related gain in body fat (24.5; SD±3.8; p<0.001) and lower among those who related loss of any body fat or lean mass (BMI 23.3 vs 24.6; p<0.001) (Table 3).

**Table 3.** Dietary intake and nutritional status of adults living with HIV according to self-perception of HAART-related body changes, São Paulo, Brazil, 2008

Nutrients and nutritional status	Any body change (gain in fat or loss in fat or lean mass)		Gain in body fat		Loss in body fat or lean mass	
	Yes	No	Yes	No	Yes	No
	Mean (SD)	Mean (SD)	Mean (SD)	Mean (SD)	Mean (SD)	Mean (SD)
Energy (kcal)	2,357.77 (960.65)	2,452.16 (987.30)	2,355.36 (959.03)	2,408.69 (977.33)	2,382.91 (979.42)	2,370.17 (951.53)
Carbohydrates†	53.77 (11.46)	54.64 (9.81)	53.85 (10.98)	55.47 (10.21)	54.71 (11.26)	54.27 (9.98)
Proteins†	17.84 (5.56)	17.62 (5.26)	18.08 (5.76)	17.52 (5.47)	17.86 (5.89)	17.84 (5.34)
Total fat†	27.66 (8.59)	27.51 (8.50)	28.08 (8.65)	27.01 (8.41)	27.43 (8.39)	27.89 (8.77)
Saturated fat†	8.71 (4.42)	8.74 (4.53)	8.87 (4.64)	8.52 (4.15)	8.61 (4.44)	8.86 (4.45)
Dietary fibre (g)$	25.23 (11.02)	27.29 (11.52)	25.29 (11.04)	26.17 (11.29)	25.55 (11.15)	25.77 (11.16)
Density (dietary fibre (g) per 1,000 kcal)	11.29 (5.18)	11.84 (5.11)	11.28 (5.22)	11.60 (5.08)	11.28 (4.97)	11.56 (5.40)
BMI‡	23.86 (3.79)	23.87 (3.90)	24.50 (3.78)	22.92 (3.66)*	23.25 (3.42)	24.61 (4.12)*

*p<0.05;

†Percentage of total energy intake;

$Adjusted by total energy intake;

BMI=Body mass index;

SD=Standard deviation

Although no associations were found in the comparisons of those who perceived and who did not perceive any body change in food intake (Table 3), men who reported gain in abdominal fat reported a lower fibre intake (25.6 g; SD±11.2 g vs 28.5 g per day; SD±12.1 g; p=0.035) (Fig.).

**Fig.**
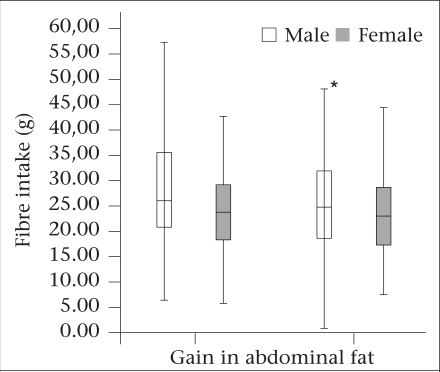
Association between energy and adjusted dietary fibre intake and self-reported gain in abdominal fat in adults living with HIV, São Paulo, Brazil, 2008

*p=0.035;
HIV=Human immunodeficiency virus

## DISCUSSION

Self-perception is one of the most-used methods to assess the HAART-related body changes (6, 17).

The prevalence of self-perceived body changes was higher in this population than that found by Santos *et al*. previously in Brazil (64.3%) (6). Tien *et al*. reported that the mean prevalence of body changes in PLHIV varied between 30% and 62% in a review study (17). The prevalence of body changes among patients living with HIV reported in the literature shows a great variation, mostly as a result of the different case definitions adopted by researchers. Santos *et al*. believe that the association between self-perceived body changes and schooling may be a result of better access to information on treatment and its implications (6).

The prevalence of self-reported body changes was significantly higher among women. This finding can perhaps be explained by the fact that women are usually more susceptible to present self-image distortions, compared to men, due to the cultural and psychosocial factors that influence and determine the construction of body image (18).

Undetectable viral load was more prevalent among those who perceived any body change, and the mean duration of HAART was also higher among this subpopulation. Some studies, based either on clinical diagnosis of lipodystrophy or on self-reported perception of body changes, found associations between the self-perceived body changes and the use of antiretroviral drugs, along with the duration of use of the nucleoside reverse transcriptase inhibitors and protease inhibitors (19, 20).

In the present study, the mean fibre intake was significantly lower among men who related gain in abdominal fat after beginning HAART-use, corroborating previous findings (21). Nonetheless, the mean fibre intake in all the subgroups was above the recommendations for the Brazilian population, which is 25 g per day (15). Hendricks *et al*. showed that persons with fat deposition were observed to consume lower amounts of total fibres, soluble fibres, insoluble fibres, and pectin (22). However, this same group did not reach the recommended dietetic fibre levels stated by the American Dietetic Association (23).

Besides studying fibre intake, the present study analyzed other nutrients, such as protein, carbohydrate, total fat, and saturated fat. All the values were within the recommendations for healthy Brazilian adults (15). It is widely known that a saturated fat-rich diet leads to hyperlipidemy and increases the risk of cardiovascular diseases (24). In this study, saturated fat intake met the guidelines—results not found by Hendricks *et al*. (22, 25).

Only schooling and BMI differed when men and women were compared. Schooling is a proxy of socioeconomic status, which may lead us to conclude that the PLHIV with lower schooling years may be facing worse life-conditions, negatively impacting self-care and adherence (26). In this population, women presented lower schooling levels than men.

Corroborating the previous finding, the prevalence of overweight (BMI >25.0) was higher than the prevalence of underweight (BMI <18.5). The prevalence of overweight in the United States has been observed among more than 50% of both men and women living with HIV (27). In a sample of 223 Brazilians living with HIV, 36.5% of women (n=19) and 28.6% of men (n=49) were considered overweight while 7.7% and 2.3% had a BMI of <18,5 (14). Furthermore, gain in body-fat was more prevalent among those with a higher BMI, and loss of fat or lean mass was more prevalent among those with a lower BMI, reinforcing the use of self-reported techniques to estimate the prevalence of body changes among PLHIV.

### Limitations

Choosing to use a 24-hour food recall can be seen as a limitation of the present study. However, it is a feasible instrument to estimate the mean values of food intake from a population group. Still, the sample-size of the present study can be seen as its strength. Care was taken when selecting individuals from different STD/AIDS Specialized Health Community Centres.

Since it was as a cross-sectional study, a further evaluation linked to the temporal framework was not possible. Despite this, it is believed that these findings can contribute to future improvements relating to the care of PLHIV.

### Conclusions

A high prevalence of self-perceived body changes has been reported in the population studied, especially in women, and an association was found between the self-perceived body changes and viral load and the duration of HAART. Similar results were observed in previous studies, contributing to building the importance of public-health policies aimed at reducing the prevalence of HAART-related body changes, providing aid in facing common issues relating to the disease and its treatment and, finally, improving the quality of life among PLHIV.

## ACKNOWLEDGEMENTS

The authors thank the City Council Program of STD and AIDS of the municipality of São Paulo for its contribution to this study. K.V. Giudici received a Junior Scientist's Scholarship from the Brazilian National Council of Scientific and Technological Development (CNPq) and A.C. Duran received a Master's Scholarship from the Research Support Foundation of the State of São Paulo (FAPESP).
